# Analysis of the national school feeding program in the municipality of Viçosa, state of Minas Gerais

**DOI:** 10.11606/S1518-8787.2018052007090

**Published:** 2018-02-07

**Authors:** Naruna Pereira Rocha, Mariana De Santis Filgueiras, Fernanda Martins de Albuquerque, Luana Cupertino Milagres, Ana Paula Pereira Castro, Mariane Alves Silva, Glauce Dias da Costa, Silvia Eloiza Priore, Juliana Farias de Novaes

**Affiliations:** I Universidade Federal de Viçosa. Programa de Pós-Graduação em Ciência da Nutrição. Viçosa, MG, Brasil; II Universidade Federal de Viçosa. Departamento de Nutrição e Saúde. Viçosa, MG, Brasil

**Keywords:** Child, School Feeding, Food and Nutrition Education, Food and Nutrition Security, Nutrition Policy, Criança, Alimentação Escolar, Educação Alimentar e Nutricional, Segurança Alimentar e Nutricional, Política Nutricional

## Abstract

**OBJECTIVE:**

To analyze the implementation of the Brazilian National School Feeding Program as a food and nutritional security policy in public schools.

**METHODS:**

This a cross-sectional study, with a quantitative and qualitative approach, carried out with 268 schoolchildren aged eight to nine years from the public school system of Viçosa, state of Minas Gerais, Brazil, in 2015. Interviews were carried out using semi-structured questionnaires with the children, parents, cooks, nutritionists, trainer of the Technical Assistance and Rural Extension Company, and president of the School Feeding Council. In order to analyze the implementation of the National School Feeding Program in Viçosa, we evaluated the direct weighing of the food served in the schools using mechanical balances with a capacity of up to 10 kg and the perception of the social players involved in the implementation of the National School Feeding Program. The children were questioned about the acceptance of and adherence to the food offered, in addition to the habit of bringing food from home. Parents reported knowledge about the School Feeding Program and Council. The qualitative analysis consisted of content analysis and quantitative analysis using the chi-square test, Fisher’s exact test, and Mann-Whitney test. We adopted the statistical significance of 5% for quantitative analysis.

**RESULTS:**

Children reported low adherence to the school feeding program and most of them used to bring food from home. Irregularities were identified in the implementation of the National School Feeding Program, such as: inadequate number of nutritionists, suspension of Council meetings, inadequate infrastructure in the areas of preparation and distribution of meals, lack of training of cooks, lack of nutritional adequacy of the food offered, and lack of actions on food and nutritional education. The Program complied with the recommendations for purchasing food from family farms.

**CONCLUSIONS:**

The National School Feeding Program presented many irregularities in Viçosa. It is important to monitor the problems identified for better reformulation and planning of the Program, in order to guarantee the food and nutritional security of the children served.

## RESUMO

**OBJETIVO:**

Analisar a execução do Programa Nacional de Alimentação Escolar como política de segurança alimentar e nutricional em escolas públicas.

**MÉTODOS:**

Estudo transversal, com abordagem quantitativa e qualitativa, realizado com 268 escolares de oito a nove anos da rede pública de ensino em Viçosa, MG, em 2015. Foram realizadas entrevistas por meio de questionários semiestruturados com as crianças, os pais, merendeiras, nutricionistas, extensionista da Empresa de Assistência Técnica e Extensão Rural e presidente do Conselho de Alimentação Escolar. Para analisar a execução do Programa Nacional de Alimentação Escolar em Viçosa, foi avaliada a pesagem direta dos alimentos servidos nas escolas, utilizando-se balanças mecânicas com capacidade de até 10 kg, e a percepção dos atores sociais envolvidos na execução do Programa Nacional de Alimentação Escolar. As crianças foram questionadas sobre a aceitação e adesão da alimentação ofertada, além do hábito de levar lanches de casa. Os pais relataram o conhecimento sobre o Programa e o Conselho de Alimentação Escolar. A análise qualitativa foi constituída pela análise de conteúdo e a quantitativa pelo Teste do Qui-quadrado, Exato de Fisher e Mann-Whitney. Adotou-se significância estatística de 5% para a análise quantitativa.

**RESULTADOS:**

As crianças relataram baixa adesão à alimentação escolar e a maior parte costumava levar lanche de casa. Foram identificadas irregularidades na execução do Programa Nacional de Alimentação Escolar, como: inadequação do número de nutricionistas, suspensão das reuniões do Conselho, inadequação da infraestrutura nas áreas de preparo e de distribuição das refeições, falta de capacitação das merendeiras, não adequação nutricional da alimentação ofertada e ausência de ações de educação alimentar e nutricional. O Programa atendeu as recomendações de compra dos alimentos provenientes da agricultura familiar.

**CONCLUSÕES:**

O Programa Nacional de Alimentação Escolar apresentou muitas irregularidades em Viçosa. É importante o monitoramento dos problemas identificados para melhor reformulação e planejamento do Programa, visando à garantia da segurança alimentar e nutricional das crianças atendidas.

## INTRODUCTION

The Brazilian National School Feeding Program (PNAE) is one of the oldest public programs of food supplementation. Its purpose is to supplement the daily nutritional needs of students enrolled in order to guarantee their Food and Nutrition Security (SAN) and contribute to the formation of good eating habits^a^. The beneficiaries of this Program are students of all basic education from public schools, philanthropic schools, and community entities in partnership with the public power^1^ ^,^ ^b^.

The PNAE is managed by the National Fund for the Development of Education (FNDE), which aims at the additional transfer of financial resources to the states, the Federal District, and municipalities^b^. In addition to the resources from the FNDE, federal entities must participate with financial consideration, maintenance of the school structures, human resources of the school feeding unit, and actions on food and nutrition education (EAN)^c^.

To implement the Program, it is important to consider some premises, such as: provision of nutritionally adequate meals, implementation of EAN actions, respect for local culture, social participation, and promotion of family agriculture (FA), contributing with the National Policy on Food and Nutrition Security (PNSAN)^1^ ^,^ ^2^.

Despite the standards and laws that guide the PNAE in the country, we found some deficiencies in its management and implementation, such as: irregularity of supply, inadequate infrastructure of schools, lack of training of the cooks, lack or inadequacy of the number of nutritionists, lack of nutritional adequacy of the food offered, among others.^3^ ^-^ ^5^ ^,^ ^d^. Thus, it is important to evaluate and identify the existing gaps to improve and maintain investments. This would facilitate the decisions made by managers and the knowledge of the implementation of all stages of the PNAE by the population^6^.

Despite the long history of the PNAE, publications on its scope and relevance are scarce^e^. Evaluations of public programs are essential to guide actions to be planned and implemented^3^ ^,^ ^d^. It is important to identify irregular situations and positive experiences to better reformulate and plan the activities of this Program.

This study aimed to analyze the execution of the PNAE as a SAN policy in public schools.

## METHODS

This is a cross-sectional study, with a qualitative and quantitative approach, carried out with children aged eight to nine years, enrolled in all public schools (n = 17) of the urban area of Viçosa, state of Minas Gerais, Brazil, in 2015.

In 2015, the municipality had seventeen public schools for children aged from eight to nine years, amounting to 1,014 children enrolled in these schools. The sample was calculated using the statistical program OpenEpi (Version 3.01). We considered the proportion of at least one resident aged under 18 years in the situation of food insecurity (23.0%) in Minas Gerais^7^, tolerable error of 5%, significance level of 5% to 15% of losses, amounting to a minimum sample size of 248 children.

The process to sample the schoolchildren was carried out in two steps. First, we carried out a stratified casual sampling in which the number of children to be sampled in each school was proportional to the total number of students in each one. Subsequently, we randomly selected the students from the seventeen public schools who belonged to the age group evaluated until completing the required number. The study had 2.5% of sample losses because some of the children did not accomplish all steps of the study. The final sample consisted of 268 students.

To analyze the execution of the PNAE in Viçosa, we evaluated: 1) direct weighing of the food served in schools, and 2) perceptions of the social players involved in the implementation of the PNAE. We interviewed the children and their parents (n = 268), a cook per school selected randomly (n = 17), all nutritionists working in the municipality and state (n = 2), the rural trainer of the Technical Assistance and Rural Extension Company of Minas Gerais (EMATER-MG)^f^, and the president of the School Feeding Council (CAE).

The interviews were carried out using a questionnaire prepared by us, with semi-structured questions, since there is no instrument to evaluate the PNAE, according to legislation. This questionnaire was tested in a pilot study with 10% of the sample.

To evaluate the meals served in the schools, we performed the direct weighing of the food using scales with a capacity of up to 10 kg. We performed it for two non-consecutive days at each school. The preparations and liquids were randomly weighed five times when distributed to the children. Researchers were present at each meal and we could obtain the recipes, ingredients, and amounts used. At the end, we considered the average weight of the meals offered in the two days^8^ ^,^ ^9^. From these records, we could calculate the supply of macronutrients and micronutrients using the software DietPro (version 5.8)^g^. This information allowed us to investigate the adequacy of the supply of 20% of the nutritional requirements recommended by the PNAE, since the students received only one meal per day^c^.

In relation to children, we evaluated the adherence, perception, satisfaction, and importance of school feeding, as well as the habit of bringing food from home. Adherence was considered satisfactory when school feeding consumption was equal to or greater than four times per week^1^ ^,^ ^5^. Perception was satisfactory when the students considered the feeding offered as “very good” or “good”^10^.

As for the parents, we investigated the knowledge about the PNAE and the CAE, the perception about school feeding, and its absence because of delays in resources or delivery of the food.

From the interviews with the cooks, we evaluated the existence of a planned menu and its realization, the number of visits of the nutritionist, and the number of trainings, as well as the improvement of the school meals with food from family agriculture (FA). The evaluation of the number of nutritionists per students was considered adequate in accordance with Resolution CFN 465/2010^h^.

We carried out on-site visits, with a checklist based on the Resolution of the Collegiate Board of Directors (RDC 216)^i^ to evaluate the conditions of storage, manipulation, preparation, and distribution of the food in the schools, according to lighting, ventilation, hygiene, and furniture conditions.

To analyze the PNAE, we conducted interviews with the professionals involved in the implementation. The reports were transcribed on the same day that the interview was conducted. The corpus of analysis of the interview with the nutritionist consisted of reports on the general data of the PNAE in the municipality and in the state, the conditions of the implementation of the Program, and the insertion of the FA.

The adequacy of the number of farmers inserted in the PNAE and the obligatory purchase of food from FA was evaluated by the reports issued by the rural trainer of the EMATER.

The reports on the performance of the CAE were obtained from an interview with the president of the board on the supervision of the actions of the PNAE, number and frequency of meetings, performance, and constitution of its structure.

After a comprehensive and exhaustive reading of the reports, the corpus for the qualitative analysis consisted of a content analysis divided into three steps according to Minayo^j^: 1) pre-analysis, 2) exploration of the material, and 3) treatment of results, inference, and interpretation. This allowed us to identify the thematic and meaning cores for the analysis^k^.

For the statistical analysis, we used the program Social Package Statistical Science (SPSS) for Windows^®^ version 20.0 (SPSS Inc., Chicago, IC, USA). The characterization of the sample was performed by the distribution of the absolute and relative frequencies and the estimates of measures of central tendency and dispersion. We used the Kolmogorov-Sminorv test to evaluate the distribution of the variables and the parametric and non-parametric tests according to their distribution. We used Fisher’s exact test and Pearson’s chi-square test to verify differences between state and municipal schools. As the variables of food consumption did not present a normal distribution, we used the Mann-Whitney test to compare the medians among children from municipal and state schools. We considered the level of statistical significance of 5%.

This study was approved by the Research Ethics Committee with Human Beings of the Universidade Federal de Viçosa (Process 663.171/2014). All parents and children were informed about the purpose of the study, and all participants signed the informed consent.

## RESULTS

We evaluated 268 schoolchildren aged from eight to nine years, being 45.5% and 54.5% of them from municipal and state schools, respectively. Of the students, 50.4% were male and there was no difference between sex and type of school.

Regarding the perception of the children on school feeding, we observed low adherence by more than half of the students (63.9%) who consumed school meals in less than or equal to three times a week. However, among those who consumed the school food more frequently, 79.4% considered it as “very good” or “good”. There was a higher prevalence of lower adherence (p = 0.02) and unsatisfactory amount served (p = 0.03) in state schools. The consumption of food not provided by the PNAE was reported by 79.5% of the students, and this habit was more prevalent in state schools (p = 0.005) (Table 1). The parents reported that they send food from home to their children because they fell “bad” for them, and they had the impression that the school food was bad and that children did not accept the food.

**Table 1:** Perception of children regarding school feeding according to the type of school. Viçosa, state of Minas Gerais, Brazil, 2015.

Variable	Total		Schools				p

			Municipal		State		
		
	n	%	n	%	n	%	
Frequency of consumption							
No	65	24.3	21	32.3	44	67.7	0.020*
≤ 3 times a week	106	39.6	48	45.3	58	54.7	
≥ 4 times a week	97	36.1	53	54.6	44	45.4	
Perception of the food							
Very good/Good	162	79.4	84	51.9	78	48.1	0.262
Regular/Bad	42	20.6	18	42.9	24	57.1	
Satisfactory amount served							
Yes	185	91.1	94	50.8	91	49.2	0.030*
No	18	8.9	7	38.9	11	61.1	
Considered as important							
Yes	231	86.2	109	47.2	122	52.8	0.172
No	37	13.8	13	35.1	24	64.9	
Food brought from home							
Never	55	20.5	33	60.0	22	40.0	0.005*
Sometimes	172	64.2	78	45.3	94	54.7	
Always	41	15.3	11	26.8	30	73.2	

* Pearson’s chi-square test.

Parents reported higher school suspension because of the absence of school feeding in municipal schools (p = 0.001). Most parents (90.3%) did not know the CAE, and this percentage was higher in state schools (p = 0.045) (Table 2).

**Table 2:** Perception of the parents regarding school feeding according to the type of school. Viçosa, state of Minas Gerais, Brazil, 2015.

Variable	Total		Schools				p

			Municipal		State		
		
	n	%	n	%	n	%	
Knowledge on the PNAE^a^							
Yes	108	40.3	52	48.2	56	51.8	0.727
No	160	59.7	70	43.8	90	56.2	
Perception of the school feeding^b^							
Positive	115	87.8	58	50.4	57	49.6	0.081
Negative	10	7.6	5	50.0	5	50.0	
Absence of school feeding^b^							
Yes	18	6.7	16	88.9	2	11.1	0.001
No	243	90.7	104	42.8	139	57.2	
Knowledge on the CAE^a^							
Yes	26	9.7	7	27.0	19	73.0	0.045
No	242	90.3	115	47.5	127	52.5	

PNAE: National School Feeding Program; CAE: School Feeding Council

^a^ Pearson’s chi-square test.

^b^ Fisher’s exact test.

Most of the schools (76.5%) did not receive technical visits from the nutritionist in the last year, and most of the cooks (70.6%) did not receive training in the last two years. State schools performed fewer training sessions for cooks in the past two years compared to municipal schools (p = 0.041).

We observed the absence of guidelines and standardization of the portions served by the cooks, as well as the absence of standard operating procedure manuals, technical data sheet, and menu in the production area. The meals were made with the mixture of all the preparations. According to the cooks, this procedure improved preparation time and increased the yield of the food.

No statistical difference was found when comparing the technical-organizational structure between municipal and state schools. Many schools presented inadequate conditions for the storage (58.8%) and preparation (58.8%) of the food (Table 3).

**Table 3:** Technical-organizational structure for the storage, preparation, and distribution of school feeding, according to the type of school. Viçosa, state of Minas Gerais, Brazil, 2015.

Variable	Total		Schools				p

			Municipal		State		
		
	n	%	n	%	n	%	
Place of storage							
Adequate	7	41.2	4	57.1	3	42.9	0.646
Inadequate^a^	10	58.8	6	60.0	4	40.0	
Presence of cafeteria							
No	10	58.8	8	80.0	2	20.0	0.052
Yes	7	41.2	2	28.6	5	71.4	
Enough furniture							
Yes	3	17.6	2	66.7	1	33.3	0.640
No	14	82.4	8	57.1	6	42.9	
Control of temperature							
Yes	-	-	-		-		-
No	17	100	10	58.8	7	41.2	
Place of preparation							
Adequate	7	41.2	5	71.4	2	28.6	0.354
Inadequate^a^	10	58.8	5	50.0	5	50.0	
Place of distribution							
Adequate	10	58.8	4	40.0	6	60.0	0.082
Inadequate^a^	7	41.2	6	85.7	1	14.3	
Conservation of furniture, walls, floors, and ceilings							
Satisfactory	10	58.8	6	60.0	4	40.0	0.640
Unsatisfactory	7	41.2	4	57.1	3	42.9	
Place to wash hands							
Yes	5	29.4	4	80.0	1	20.0	0.278
No	12	70.6	6	50.0	6	50.0	
Hygiene of the area of production and distribution							
Satisfactory	13	76.5	6	46.2	7	53.8	0.088
Unsatisfactory	4	23.5	4	100	-	-	
Exposure of the menu							
Yes	9	52.9	1	20.0	4	80.0	
No	8	47.1	9	75.0	3	25.0	0.581

^a^ Lighting/ventilation/hygiene.

All analyzes were performed by Fisher’s exact test.

No school met the reference value of the PNAE for macronutrients and micronutrients, except for magnesium and vitamin A in state schools (Table 4).

**Table 4:** Median, minimum, and maximum values for energy and nutrients of the menus offered in the school feeding of municipal and state public schools. Viçosa, state of Minas Gerais, Brazil, 2015.

Energy and nutrients	PNAE*	Municipal schools		State schools		
		
	6 to 10 years	Median	Min–Max	Median	Min–Max	p
Energy (kcal)	300	207.58	81.30–427.60	202.20	162.20–284.60	0.558
Carbohydrate (g)	48.8	36.51	15.60–69.80	32.00	20.60–44.60	0.329
Protein (g)	9.4	7.16	1.70–14.10	7.90	4.60–16.70	0.354
Lipids (g)	7.5	4.71	1.30–13.40	4.40	1.88–13.30	0.845
Fiber (g)	5.4	3.02	0.70–10.34	2.47	1.40–6.30	0.695
Vitamin A (ug)	100	63.45	15.00–203.80	118.80	0.0–20210	0.283
Vitamin C (mg)	7.0	2.46	0.60–16.40	1.60	0.00–4.50	0.407
Calcium (mg)	210	52.85	7.20–203.30	21.20	17.58–38.10	0.079
Iron (mg)	1.8	1.70	0.20–2.70	1.00	0.80–1.70	0.184
Zinc (mg)	1.3	1.00	0.30–1.50	1.00	0.60–2.20	0.118
Magnesium (mg)	37	28.20	17.10–93.90	52.90	29.10–64.60	0.728

PNAE: National School Feeding Program; Min: minimum; Max: maximum

* Reference of the recommendations that establishes the minimum offer of 20% of daily nutritional needs when a meal is offered for students who study part time, according to Resolution 026/2013.

All analyzes were performed by Mann-Whitney test.

The municipality of Viçosa had only one nutritionist as the technician responsible for the PNAE for the ten urban municipal schools at the time of the study. However, after four months performing this study, the professional was reassigned to the health service, and the PNAE was left without any nutritionist. Only one nutritionist was responsible for coordinating the school feeding actions of approximately 2,017,474 students in the State of Minas Gerais at the time of the study.

According to the nutritionist of the municipality, the products received were not sufficient for the elaboration of a balanced menu for school feeding. They could not always meet all the established regulations, such as the minimum provision of three servings of fruit per week. The fresh food planned for the menu were mostly supplied by local producers, cooperatives, or associations, and the other types of food were acquired from public bidding by the local government. The food had satisfactory quality and hygienic-sanitary conditions and there was an increase in the participation of FA. This allowed the meeting of the demand and improved the quality of the products, as well as the training and performance of the CAE in Viçosa. However, all kitchens in municipal schools were domestic-sized and did not have important equipment such as scales, thermometers, and blenders.

According to the perception of the nutritionist of the municipality, the PNAE presented some irregularities, such as: 1) the need for more nutritionists to meet local demand, 2) the lack of training of cooks and periodic visits to schools, 3) the lack of equipment for the nutritional evaluation of schoolchildren, 4) the shortage of educational materials to work on the formation of eating habits, and 5) the lack of vegetable gardens in schools.

The nutritionist responsible for the state schools reported advances in the implementation of the PNAE, such as the technical supervision of approximately sixty state schools by seven nutritionists. In this way, actions of technical supervision and guidance could be carried out at the state schools and there was an increase in the acquisition of food from FA. However, this action occurred until June 2015, and only one nutritionist remained for the state technical supervision of the PNAE at the time of the study.

According to the rural trainer of the EMATER, the number of family farmers was sufficient to meet the PNAE in Viçosa, but they had difficulties. Many farmers did not have transportation to facilitate delivery on the established days and times. In addition, the delivery of the products was not always good, given the lack of training and adequate tools for the transport of the food.

The meetings of the CAE were held monthly with the participation of twelve members (nutritionist, president of the CAE, person responsible for the delivery of food, parents, teachers, and directors) and had the purpose of visiting schools and supervising them in relation to the application of resources, the execution of the menus, the quality of the food, the good practices of the cooks, and other functions. However, after the nutritionist left the PNAE, these meetings were temporarily suspended.

The difficulties reported by the president of the CAE were: precariousness of the physical structure of schools, logistical difficulty for weekly delivery of food, resistance of the cooks in relation to good manipulation practices, and difficulty in meeting the suggestions for modifications in the physical structure of schools and in the menus in the short term. The president considered it necessary to include more fruits on the menu.

## DISCUSSION

The PNAE presented irregularities in Viçosa, such as: inadequacies in the number of nutritionists, suspension of the meetings of the CAE, inadequate infrastructure in the areas of preparation and distribution of meals, lack of training of cooks, low adherence to school feeding, lack of EAN actions, and lack of nutritional adequacy of the food offered.

State schools had higher prevalence of inadequacies in the implementation of the PNAE in relation to municipal schools. The infrequent supervision of the nutritionist in schools, especially in state schools, may have contributed with the higher prevalence of the lack of knowledge about the CAE by the parents, the lack of training of the cooks, and the habit of parents of sending food from home to the child to replace the school meal. However, municipal schools showed a greater lack of school feeding.

Resolution FNDE/CD 32/2006^l^ establishes the work and obligatoriness of the nutritionist in the implementation of the PNAE and resolution CFN 465/2010^h^ recommends that the enrollment of this professional must meet the current minimum professional parameters and the appropriate workload, based on the number of students of the Program in each region. As the technician responsible for the PNAE, the nutritionist assumes an important role in the elaboration of the school menu, in the orientation to choose the types of food, and in the evaluation of the quality of the food to be used^11^ ^,^ ^m^.

In relation to the CAE, it did not present activity at the time of this study. The CAE is an important ally in the monitoring of the guidelines of the PNAE with the role of carrying out social control, thus allowing better efficiency and the reach of the SAN of the schoolchildren^2^. It constitutes an important space for participation and social control^12^. In addition, it is a space that needs to be conquered and known by the parents who participated in this study.

Regarding food handlers and the quality of the food purchased, Resolution 26/2013^c^ of the PNAE states that they must comply with the legislation established by the National Agency of Health Surveillance (ANVISA). In addition to the provision of a healthy and varied diet, the cooks must undergo training on the hygienic-sanitary conditions of production, in which the structure of the schools is fundamental for the hygiene of the food and handler^13^.

Another aspect to be worked on is the habit of parents of sending food with their children. Flores et al.^d^ highlight the ignorance of the context and objectives of the PNAE by parents and students. Generally, knowledge is limited to food itself and not to the social commitment of the PNAE linked to the SAN axes^d^.

More than half of the schools did not have a space reserved for the cafeteria and adequate furniture, in addition to the absence of gardens. The presence of adequate cafeteria and furniture is important for a meal environment that is pleasurable, quiet, and conducive to a healthy social life. This gives schoolchildren greater awareness of the act of eating^5^. A space planned for gardens could be used for the cultivation of vegetables that could be used in the school itself^d^.

The shortage of educational materials and the lack of periodic visits from the nutritionist to schools hinder the EAN actions, the formation of healthy eating habits, and the improvement of the acceptance of new food. It is essential to incorporate educational activities related to nutrition and health into education^4^.

Several Brazilian municipalities have irregularities in the implementation of the PNAE. Gabriel et al*.* ^3^ have found inadequacy in the number of nutritionists and insufficient nutritional assessment equipment, educational materials, and computer software in the municipalities of Santa Catarina, Brazil. Flores et al.^d^ have observed the need for improvements in the kitchen and cafeteria facilities, as well as the lack of training of the cooks in Araraquara, State of São Paulo, Brazil.

Adherence to school feeding was low in more than half of the students. Similar results have been found by other studies that show low acceptance and adherence in the school environment^4^ ^,^ ^5^ ^,^ ^10^. Some factors such as the lack of EAN, inadequate eating habits, and unpleasant taste of the preparations may be related to this situation^4^.

Some measures to increase knowledge on the PNAE and to ensure greater adherence and acceptance of school meals can be taken. Among them, we can mention the implementation of EAN actions developed with the participation of schoolchildren, teachers, parents, and local community in actions that encourage the planting of gardens, the organization of food fairs and activities that stimulate a healthy lifestyle, the revision of food distribution schedules, and the guarantee of adequate infrastructure and human resources^5^ ^,^ ^14^.

There was a lower supply of macronutrients and micronutrients in school meals compared to what is recommended. The adequate intake of energy, macronutrients, and micronutrients are required for the growth, cognitive development, and immune status of schoolchildren^15^. Some nutritional deficiencies tend to be more prevalent among the most vulnerable populations, but they may also be present in children with inadequate eating habits^n^.

Actions to promote the adequacy of the implementation of the PNAE are important. For many children, the school meal is the most complete meal or the only meal of the day. It provides energy and micronutrients that are often not supplied in regular amounts in the daily diet to meet the needs of schoolchildren^16^.

One of the positive points of the PNAE in Viçosa was the meeting of the 30% of food expenses with family agriculture. This benefit enables the construction of markets, the formation of cooperatives and organizations, and the reduction of rural exodus^17^. The stimulus and the support to FA become relevant for the actions of the SAN and local development^18^.

This was the first study to evaluate school feeding in all urban public schools in Viçosa, as well as the perception of the social players involved in the implementation of the PNAE. As this Program has an important contribution to the PNSAN, the nutritional evaluation of the school meal becomes of utmost importance. Many schoolchildren approach the pubertal spurt period, in which every effort must be guaranteed for the expression of the growth potential and development.

We could not carry out the evaluation of the PNAE in Viçosa, since there is no instrument for this purpose, according to legislation. New studies are needed for the construction of instruments to evaluate this Program.

The PNAE in the city of Viçosa presented several irregularities, such as inadequacies in the number of nutritionists, suspension of the meetings of the CAE, inadequate infrastructure in the areas of preparation and distribution of meals, lack of training of cooks, lack of EAN actions, and lack of nutritional adequacy of the food offered. State schools had more irregularities in the implementation of the PNAE in relation to municipal schools. One of the positive points of the PNAE was the fulfillment of the requirement to meet the 30% of food expenses with family agriculture.

Constant monitoring is necessary to identify irregularities in the Program. The nutritionist is fundamental in the execution and the guarantee that objectives are met. The state and municipality should improve the quality of school meals, since the meals offered did not meet the nutritional needs established by the PNAE, in addition to the low adherence to school feeding. The works of the EAN with children, parents, and teachers are important to promote healthy eating habits, as well as to raise awareness and appreciate the PNAE as one of the fundamental axes for guaranteeing the SAN of the children.

## Footnotes

a FNDE- Fundo Nacional de Desenvolvimento da Educação. Brasília (DF): MEC; s.d. [cited 2016 Oct 14]. Available from: http://www.fnde.org.br

b FNDE- Fundo Nacional de Desenvolvimento da Educação Relatório de atividades. Brasília (DF): MEC; 2014 [cited 2015 Jul 4]. Available from em: http://www.fnde.gov.br/Programas/Merenda

c Ministério da Educação (BR), Fundo Nacional de Desenvolvimento da Educação (FNDE). Resolução nº 26, de 17 de junho de 2013. Dispõe sobre o atendimento da alimentação escolar aos alunos da educação básica no âmbito do Programa Nacional de Alimentação Escolar - PNAE. *Diario Oficial Uniao*. 18 jun 2013; Seção 1.

d Flores TMC, Almeida LMMC, Ferrante VLSB. Programa Nacional de Alimentação Escolar no município de Araraquara-SP. *Rev Pol Publicas*. 2014;18(1):281-92. https://doi.org/10.18764/2178-2865.v18n1p281-292

e Castro TG, Matos ELC, Leite MS, Conde WL, Schuch I, Veiga J, Zuchinali P, et al. Características de gestão, funcionamento e cardápios do Programa Nacional de Alimentação Escolar em escolas Kaingáng do Rio Grande do Sul, Brasil. *Cad Saude Publica*. 2014;30(11):2401-12. https://doi.org/10.1590/0102-311X00215513

f The role of the social welfare trainer is to advise farmers and their organizations on agricultural and non-agricultural activities and on improving their living conditions, benefiting the provision of healthy food for the school feeding program. Empresa de Assistência Técnica e Extensão Rural do Estado de Minas Gerais. Extensionista do bem estar social. Belo Horizonte; s.d. [cited 2016 Nov 1]. Available from: http://www.emater.mg.gov.br/

g Bressan J, Esteves E. Sistema de suporte de avaliação nutricional e avaliação de dietas - DietPro [computer programmer]. Versão 5.8. Belo Horizonte: Agromídia Software; 2001.

h Conselho Federal de Nutricionistas. Resolução CFN Nº 465/2010. Dispõe sobre as atribuições do nutricionista, estabelece parâmetros numéricos mínimos de referência no âmbito do Programa de Alimentação Escolar (PAE) e dá outras providências. *Diario Oficial Uniao*. 25 ago 2010 [cited 2017 Jul 31]; Seção 1:118-9. Available from: http://www.cfn.org.br/novosite/arquivos/resol-cfn-465-atribuicao-nutricionista-pae.pdf

i Agência Nacional de Vigilância Sanitária. RDC nº 216, de 15 de setembro de 2004. Dispõe sobre regulamento técnico de boas práticas para serviços de alimentação. Brasília (DF): ANVISA; 2004 [cited 2017 Jul 31]. Available from: http://portal.anvisa.gov.br/ documents/33916/388704 /RESOLU%25C3%2587% 25C3%2583O-RDC%2BN %2B216%2BDE%2B15%2 BDE%2BSETEMBRO%2BD E%2B2004.pdf/23701496-925d -4d4d-99aa-9d479b316c4b

j Minayo MCS. O desafio do conhecimento: pesquisa qualitativa em saúde. 10.ed. São Paulo: Hucitec/Abrasco; 2007.

k Bardin L. Análise de conteúdo. São Paulo: Edições 70; 2011.

l Ministério da Educação (BR), Fundo Nacional de Desenvolvimento da Educação, Conselho Deliberativo. Resolução FNDE/CD Nº 32 de 10 de agosto de 2006. Estabelecer as normas para a execução do Programa Nacional de Alimentação Escolar - PNAE. *Diario Oficial Uniao*. 11 ago 2006; Seção 1:27.

m Ministério da Educação (BR), Fundo Nacional de Desenvolvimento da Educação, Secretaria de Educação a Distância. Programa Nacional de Formação Continuada a Distância nas Ações do FNDE. Formação pela Escola. 2.ed. atual. Brasília (DF): MEC, FNDE, SEED, 2008.

n Ministério da Educação (BR), Fundo Nacional de Desenvolvimento da Educação. Manual de orientação para a alimentação escolar na educação infantil, ensino fundamental, ensino médio e na educação de jovens e adultos. 2.ed. Brasília (DF): PNAE: CECANE-SC; 2012.
